# Enhanced Anti-cancer Potency Using a Combination of Oleanolic Acid and Maslinic Acid to Control Treatment Resistance in Breast Cancer

**DOI:** 10.34172/apb.2023.057

**Published:** 2022-09-18

**Authors:** Cigir Biray Avcı, Fatma Sogutlu, Neslihan Pinar Ozates, Behrouz Shademan, Cumhur Gunduz

**Affiliations:** Department of Medical Biology, Faculty of Medicine, Ege University, Izmir, Turkey.

**Keywords:** Breast cancer, mTOR signal pathway, Therapeutic resistance, Oleanolic acid, Maslinic acid

## Abstract

**Purpose:**

The phosphatidylinositol 3-kinase/AKT/mammalian target of rapamycin (PI3K/AKT/ mTOR) pathway is a complex intracellular metabolic pathway that leads to cell growth and tumor proliferation and plays a key role in drug resistance in breast cancer. Therefore, the anti-cancer effects of oleanolic acid (OA), maslinic acid (MA), and their combination were investigated to improve the performance of the treatment strategy.

**Methods:**

We investigated the effect of OA and MA on cell viability using the WST-1 method. The synergistic effect of the combination was analyzed by isobologram analysis. In addition, the effects of the two compounds, individually and in combination, on apoptosis, autophagy, and the cell cycle were investigated in MCF7 cells. In addition, changes in the expression of PI3K/AKT/mTOR genes involved in apoptosis, cell cycle and metabolism were determined by quantitative RT-PCR.

**Results:**

MA, OA, and a combination of both caused G0/G1 arrest. Apoptosis also increased in all treated groups. The autophagosomal LC3-II formation was induced 1.74-fold in the MA-treated group and 3.25-fold in the MA-OA-treated group. The combination treatment resulted in increased expression of genes such as GSK3B, PTEN, CDKN1B and FOXO3 and decreased expression of IGF1, PRKCB and AKT3 genes.

**Conclusion:**

The results showed that the combination of these two substances showed the highest synergistic effect at the lowest dose and using MA-OA caused cancer cells to undergo apoptosis. The use of combination drugs may reduce the resistance of cancer cells to treatment.

## Introduction

 Breast cancer is one of the most common cancers that cause death in women.^1^ Breast cancer is classified into molecular subclasses based on hormone receptor status and epidermal growth factor 2 (HER2) status. Lumen A (ER + , HER2-) breast cancer is one of the most common types.^2^ The PI3K/Akt/mTOR pathway is essential in mammalian cells that control growth through growth stimuli. Factors such as increased growth factors, mutations, loss of gene function, or activation of kinase genes cause inhibition of the pathway leading to carcinogenesis.^3,4^ The PI3K/Akt/mTOR pathway has been identified as a cancer resistance pathway.^5,6^ In patients receiving hormone therapy for breast cancer, the PI3K/Akt/mTOR pathway has been identified as an escape pathway that causes tumor cells to become resistant to treatment.^7^ Cancer cells use a variety of strategies to overcome the immune response of the body’s antitumor system. Therefore, the PI3K/Akt/mTOR pathway can be a target for chemotherapy. However, the potential of natural products as a specific drug is different and using natural substances in combination has a significant effect and minimizes the toxicity and side effects of chemotherapeutic drugs.^8,9^

 The oleanolic acid (OA) and maslinic acid (MA), found in over 1600 plant species, have been included in the pentacyclic triterpenes of the Olean type group. *Olea europaea* is the primary source of both natural compounds. OA (31-44%) and MA (55-68%) were found mainly in the intracuticular part of the olive. Besides biological activities such as antiviral, anti-inflammatory, antidiabetic, antimicrobial, and cardioprotective properties with a proven pharmacological safety profile, have anti-cancer properties.^10,11^

 In the studies conducted, the efficacy of OA on malignant gliomas and hematological malignancies in breast, hepatocellular, lung, colon, pancreatic, and gallbladder cancers were demonstrated and found to affect cell survival via PI3K/Akt/mTOR, NF-KB, TGF-β, ERK/JNK/p38 MAPK, signaling pathways.^12,13^ The anti-cancer effects of MA on melanoma, colon, ovarian, lung, oral carcinoma, liver, and breast cancers were investigated and found to have antiproliferative, cytostatic, apoptotic, and autophagic effects via JAK/STAT3, AMPK/mTOR, p38 MAPK, and NF-KB signaling pathways.^14-16^ This study aimed to determine the effects of OA and MA on toxicity, apoptosis, and autophagy in the MCF7 cell line, both individually and in combination. In addition, changes in the expression of PI3K / AKT / mTOR genes involved in apoptosis, cell cycle, and metabolism were determined by quantitative RT-PCR.

## Materials and Methods

###  Cell line and culture conditions

 The MCF7 cell line was purchased from the American Type Culture Collection (ATCC# HTB-22^TM^). Cells were cultured in DMEM (Cat. No. 01-052-1A, Biological Industries, USA) containing 2 mM L-glutamine, 1% penicillin-streptomycin and 10% FBS in an incubator with 95% humidity, 37°C and 5% CO_2_. The cultured cells were examined with an “inverted” microscope. OA (cat. no.: O5504) and MAs (cat. no.: M6699) were purchased from Sigma-Aldrich. OA and MA were soluble in DMSO at 44 mM and 10 mM, respectively, and were stored diluted at–20°C. The subsequent dilutions were prepared in DMEM.

###  Cell viability assay

 Cells were seeded in a 96-well plate (6.2 × 10^3^ cells per well) and incubated for one day under standard conditions. MCF7 cancer cells were treated with different concentrations of OA (462-184.8 μM) and MA (100-40 μM) for 48 and 72 hours. After this period, the cytotoxic effect of OA, MA, and their combination on the MCF7 cell line was quantified using the cell proliferation reagent WST-1 (Cat. No.: 11644807001, Roche). WST-1 (10 μL/well) solution was added and incubated for 1 hour. Optical density (OD) was measured every 15 minutes after incubation using a microplate reader (Multiskan FC, Thermo) in the reference range of 450 nm absorbance 620 nm. IC50 doses that reduced cell proliferation by 50% were determined for OA and MA in the MCF7 cell line.

###  Isobologram

 In the CalcuSyn 2.0 software, in which the Chou-Talalay theorem based on the median effect equation was used, whether the OA and MA combinations showed synergistic, antagonistic or additive effects were automatically analyzed by using percent cytotoxicity values. The combination ratio of MA: OA was 100:462, respectively. The dose-effect curve was plotted, the fractions (Fa) affected by the administered doses, the doses ED50 (effective dose for 50% of the population), the combination indices (CI) and dose reduction indices (DRI) of these doses and the slope (m) of the dose-effect curve was determined.^17^

###  Cell cycle assay 

 The effect of OA and MA on different stages of the cell cycle was determined by flow cytometry. Briefly, cells (3.5 × 10^5^) were seeded into a 6-well plate and allowed to adhere for 24 hours. Then, 72 hours after treatment with OA, MA, and MA-OA, cells were removed with trypsin and centrifuged at 400 × g for 5 minutes. The cells were rinsed twice with 1 × PBS. According to the protocol of the kit (cat. no. 340242, BD Biosciences), cells were washed twice with cell cycle buffer. Solutions A, B, and C were added to each. The cells were analyzed by flow cytometry (Becton-Dickinson, USA). The group not treated with natural substances served as controls.

###  Apoptosis assay 

 Evaluation of the apoptotic effect of OA and MA on MCF7 cell lines through the phosphatidylserine (PS) in the cell membrane was performed according to the manual of the FITC Annexin V Apoptosis Detection Kit (Cat. No.: 556547, BD Pharmingen, USA) protocol by counting 3.5 × 105 cells on the BD ACCURI C6 flow cytometer (BD Biosciences Pharmingen). After OA and MA were added to the cells at the desired dosage, their apoptotic effect was examined for 72 hours. The group that was not treated with OA and MA was considered as the control group.

###  Autophagy assay 

 The Premo Autophagy Tb/GFP TR-FRET LC3B Expression Kit (cat. no. A14070; Invitrogen; USA) was used to determine the effect of OA, MA, and MA-OA on autophagosome formation in MCF7 cells. Cells supplemented with Optimum I with 10% serum were mixed with different concentrations of LC3B-GFP BacMam and allowed to attach in a 96-well plate. After 24 hours, the IC50 values of the natural compounds and the ED50 values of the combination were applied to the cells of the dose group. In the negative control group, chloroquine was added to the cells to inhibit lysosome activity by accumulating LC3B II. The growth medium was also added to the cells of the control group. Cells were incubated at room temperature for 2 hours after adding lysis buffer to each well of the 96-well plate. TR-FRET emission was measured using the 332 nm excitation, 518 nm, and 488 nm emission filters in the Varioskan Flash Multimode Microplate Reader (Thermo Scientific).

###  Gene expression 

 The MCF7 cell line was treated with OA and MA for 72h to examine the expression of AKT3, BAD, EIF2AK2, GSK3β, FOXO3, PRKCB, PTEN, TLR4, CDKN1B genes by quantitative real-time PCR (qRT-PCR). In brief, total RNA was extracted from control and treated cells using the RNeasy Plus Mini Kit (Cat. No. 74134, Qiagen, USA) according to the manufacturer’s instructions. The quantity and quality of extracted RNA were assessed by Nanodrop and electrophoresis on a 1% agarose gel. The RT2 First Strand Kit (cat. no. 330401; Qiagen, USA) was used for cDNA synthesis. The specific primers are listed in Table 1. The GAPDH gene was considered an endogenous control gene for normalization of the AKT3, BAD, EIF2AK2, GSK3β, FOXO3, PRKCB, PTEN, TLR4, and CDKN1B genes. The threshold (Ct) was determined for the genes. The comparative 2^-ΔΔCt^ threshold cycle method was used for data analysis.

**Table 1:** Primer sequences for the genes studied

**Gene**	**Primer sequence**	
AKT3	Forward	5′-TGGACAAAGATGGCCACATA-3′
	Reverse	5′-ATCAAGAGCCCTGAAAGCAA-3′
BAD	Forward	5'-ACCCGGCAGACAGATGAG-3'
	Reverse	5'-CTTCCTCTCCCACCGTAGC-3'
EIF2AK2	Forward	5'-AAAAATCAGGAGACCCTGGCTA-3'
	Reverse	5'-TCTTCCCGTATCCTGGTTGGA-3'
FOXO3	Forward	5′-ACGGTGTTCGGACCTTCATC -3′
	Reverse	5′-TGCTGGCCTGAGACATCAAG-3′
GSK3B	Forward	5'-GGAACTCCAACAAGGGAGCA-3'
	Reverse	5’-TTCGGGGTCGGAAGACCTT A-3’
PRKCB	Forward	5'-GCCTACCCCAAGGTCCATGT-3'
	Reverse	5'-CTTGGTCATGAGCCCTTTG-3'
PTEN	Forward	5′-ACCAGGACCAGAGGAAACCT-3′
	Reverse	5′-GCTAGCCTCTGGATTTGACG-3′
TLR4	Forward	5′-CACTGTTCTTCTCCTGCCTGAC-3′
	Reverse	5′-TGG TTGAAGAAGGAATGTCATC-3′
CDKN1B	Forward	5’- GGGTAGAGTTGGGGGTAG-3’
	Reverse	5’-ACAAACCTACTCTAACTAACCT-3’
IGF1	Forward	5’- GGCATAGCTGGCCAAACAA-3’
	Reverse	5’- CACTTGGGAGAAGGCTTAGAATAAA-3’
GAPDH	Forward	5'-CTGACTTCAACAGCGACACC-3'
	Reverse	5'-TAGCCAAATTCGTTGTCATACC-3'

###  Statistical analysis

 Student t-test was used for statistical analysis of gene expression. A one-way ANOVA was performed for analysis of autophagy results. IC50 values were calculated using CalcuSyn v.2.1 software. The *P* < 0.05 was considered statistically significant.

## Results

###  Evaluation of cell viability and combination of OA and MA

**Figure 1:**
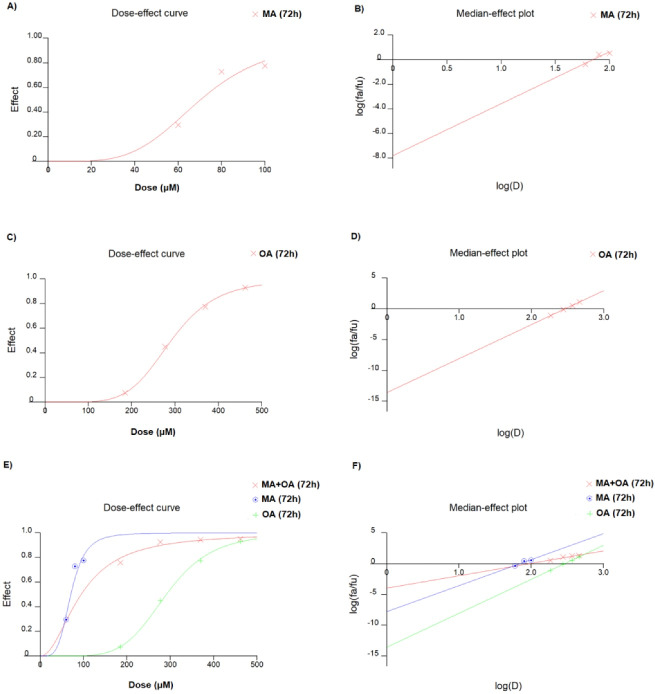
Dose-response and Median-effect graph of A, B) MA, C, D) OA and, E, F) their combination in MCF7 for 72 h

**Figure 2:**
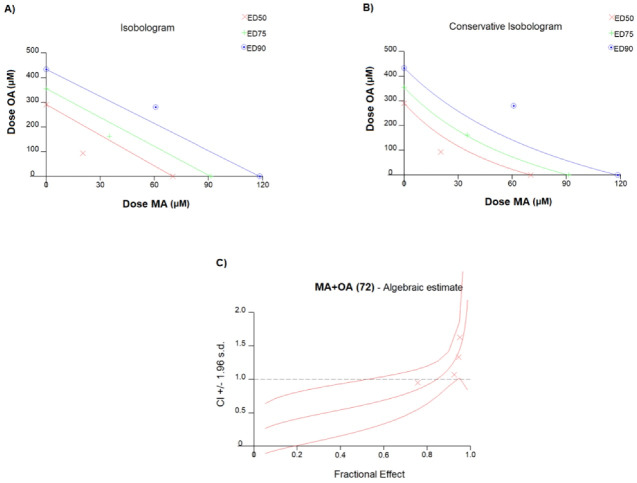
Representation of synergistic, additive and antagonistic effect of ED50, ED75 and ED9 value in A) isobologram graph and B) conservative isobologram, C) Algebraic estimation graph showing combination index corresponding to fractions

 In MCF7 cells, the IC50 values of OA and MA were 70.3 μM (r: 0.9994, m: 5.50685 + /- 0.128892) and 291 μM (r: 0.94200, m: 4.21410 + /- 1.501392), respectively (Figure 1). The Combination index of the fractions affected at 50% (ED50), 75% (ED75), and 90% (ED90) were 0.612, 0.842, and 1.159, respectively, and the experiments were continued with the ED50 value that showed the highest “synergistic effect” (Figure 2). The ED50 value of MA and OA was detected to be 20.34 μM with 3.45 DRI and 94.01 μM with 3.09 DRI, respectively (r: 0.94336, m: 2.01039 + /- 0.499948).

###  Evaluation of apoptosis

**Figure 3:**
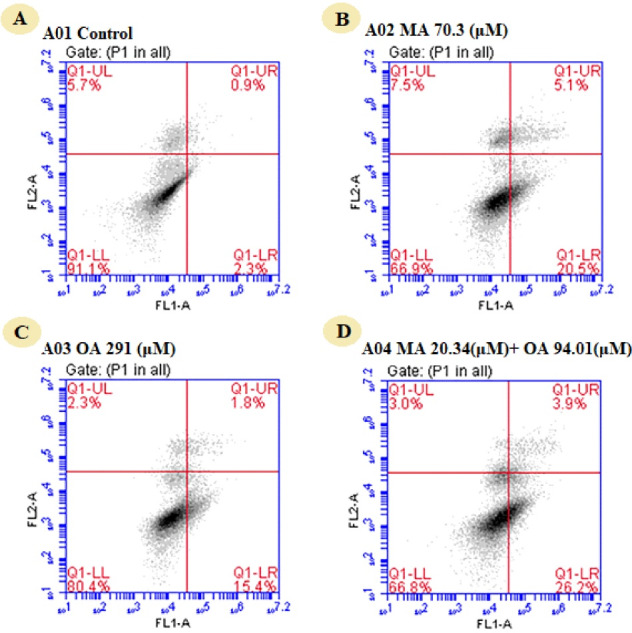
Assessment of apoptosis by annexin V method in MCF7 cells treated with OA, MA, and MA-OA. (A) Control group, untreated, (B) MA (70.3 μM), (C) OA (291 μM), (D) The combination of MA (20.34 μM) and OA (94.01 μM)

 When apoptosis was assessed by the annexin V method, apoptosis was 3.1% in the untreated group, 25.6% in the group treated with the IC50 value of MA, and 17.2% in the group treated with the IC50 value of OA. Apoptosis in the 50% affected fraction of the two natural compounds was 30.1%. In the breast cancer model, OA, MA, and the combination induced apoptosis by 8.25-fold, 5.59-fold, and 9.7-fold, respectively (Figure 3).

###  Evaluation of autophagy

**Figure 4:**
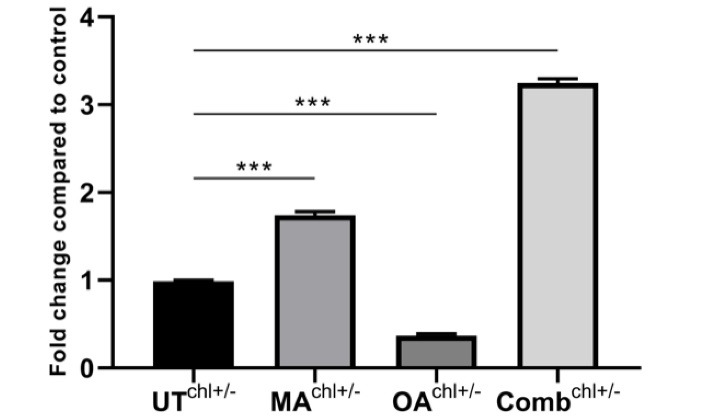
To measure the amount of autophagosome formation from GFP-labeled LC3B-expressing cells using a TR-FRET immunoassay approach. Determining autophagosome formation in the control group with MA, oleanolic acid and the combination group as a fold change. (**A)** (The 1.74-fold increase) MA (70.3 μM), (**B)** (the 0.37-fold increase) OA (291 μM), (**C)** (the 3.25-fold increase) the combination of MA (20.34 μM) and OA (94.01 μM). ***P < 0.001 for all by one-way ANOVA

 The effect of IC50 values of the natural compounds and ED50 value of the combination on autophagy was compared with the untreated group. Autophagosomal LC3-II formation was induced 1.74-fold in the group treated with MA, 0.37-fold in the group treated with OA, and 3.25-fold in the combination group (Figure 4).

###  Evaluation of cell cycle 

**Figure 5:**
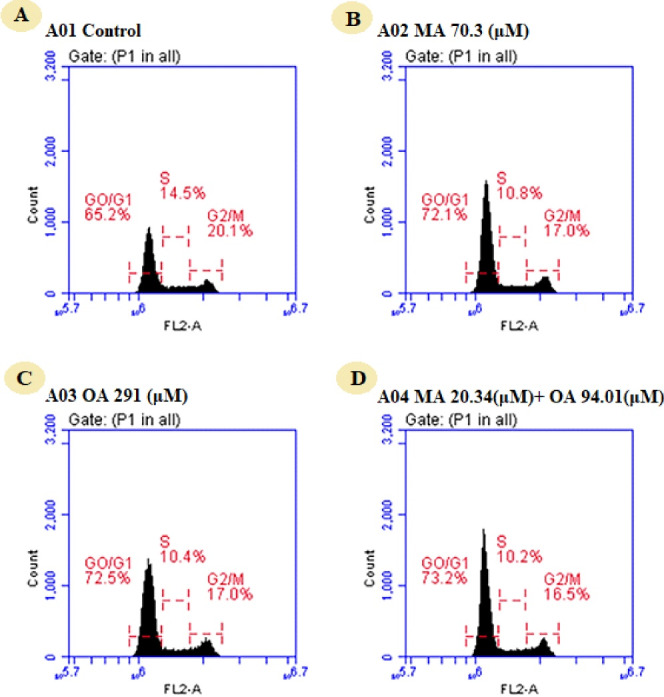
Assessment of Cell cycle by flow cytometry in MCF7 cells treated with OA, MA, and MA-OA. **A) **Control group, untreated (**B)** MA (70.3 μM), (**C)** OA (291 μM), (**D) **Thecombination of MA (20.34 μM) and OA (94.01 μM)

 When the effect of the natural compounds and the combination on the cell cycle was studied in MCF7 cells, it was found that the cell population was 65.2% in G0/G1 phase, 14.5% in the S phase and 20.1% in the G2/M phase. The cell population in G0/G1 phase was increased to 72.1% by MA, 72.5% by OA, and 73.2% by the combination treatment. It was found that the natural compounds and the combination caused G0/G1 arrest (Figure 5).

###  Evaluation of genes involved in PI3K/AKT/mTOR signaling pathway 

**Figure 6:**
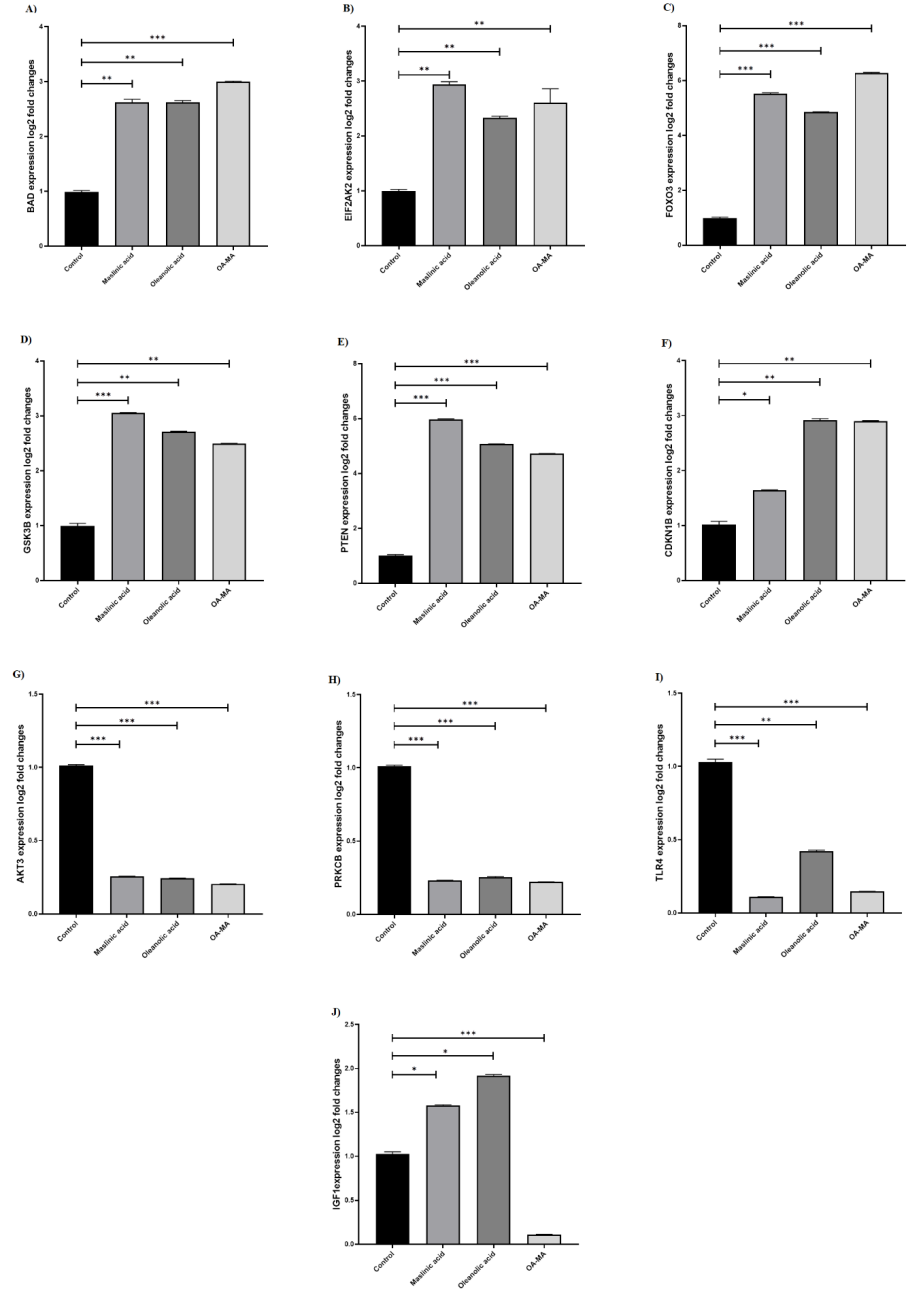
Changes in gene expression BAD (A), EIF2AK2 (B), FOXO3 (C), GSK3B (D), PTEN (E), CDKN1B (F), AKT3 (G), PRKCB (H), TLR4 (I), and IGF1 (J) in MCF7 cell line treated with MA, Oleanolic acid, and MA-OA

 The effect of OA, MA, and their combination on the expression of genes involved in the PI3K/AKT/mTOR signaling pathway in MCF7 cells was investigated. It was found that the expression of BAD, EIF2AK2, FOXO3, GSK3β, PTEN, CDKN1B, GSK3β genes after MA increased 2.66-fold, 2.94-fold, 5.52-fold, 3.06-fold, 5.98-fold, 1.64-fold, 3.06-fold, and the AKT3, PRKCB, and TLR4 genes decreased 3.89-fold, 4.32-fold, 9.04-fold, respectively. The expression of BAD, EIF2AK2, FOXO3, GSK3β, PTEN, CDKN1B, GSK3β genes after OA treatment increased 2.61-fold, 2.36-fold, 4.85-fold, 2.70-fold, 5.05-fold, 2.90-fold, 2.70-fold and AKT3, PRKCB, TLR4 genes decreased 4.11-fold, 3.92-fold, 2.36-fold, respectively. In the combination-treated MCF7 cells, the expression of BAD, EIF2AK2, FOXO3, GSK3β, PTEN, CDKN1B, and GSK3β genes was observed to increase 3.01-fold, 2.46-fold, 6.27-fold, 2.49-fold, 4.71-fold, 2.89-fold, 2.49-fold, respectively. AKT3, PRKCB, TLR4, IGF1 genes down-regulated 4.88-fold, 4.54-fold, 6.88-fold, 8.96-fold, respectively (Figure 6).

## Discussion

 Natural and synthetic compounds are used as chemotherapeutic agents and are one of the treatment options in cancer treatment.^18,19^ However, the toxic side effects of synthetic chemotherapeutic agents and the stringent approval procedures before administering them to patients have led researchers to focus on natural compounds to provide safer alternatives for cancer treatment and shorten the drug approval time.^20^ In our study on MCF7 cells, the IC50 values of OA and MA were 70.3 μM and 291 μM, respectively. The Combination index of the fractions affected at ED50, ED75, and ED90 were 0.612, 0.842, and 1.159, respectively, and the experiments were continued with the ED50 value that showed the highest “synergistic effect”. When OA and MA were used separately and in combination in the MCF7 cell line, it was found that the combination therapy showed a significant synergistic effect. In addition, a dose reduction was observed when the two cases were combined compared with a single treatment. We speculate that the synergistic effect of the combination and the increase in dose reduction index may be related to the distal/proximal targeting of the PI3K/AKT/mTOR pathway with OA and MA.

 The studies conducted have shown the therapeutic potential of many phytochemicals against cancer. The triterpenoids found in rarely consumed fruits, vegetables, and plants, representing the group of phytochemicals, and belonging to the oleanane-type terpenoid group, are easy to find and inexpensive. They also show that their chemo preventive and antitumor activities pave the way for their use as natural anti-cancer agents.^21^ Awareness of the important role of the PI3K/Akt/mTOR pathway in breast cancer survival and proliferation and evidence that the oleanane derivatives OA and MA target the PI3K/Akt/mTOR pathway have been found in attractive breast cancer treatments.^16^ Awareness of the important role of the PI3K/Akt/mTOR pathway in breast cancer survival and proliferation and evidence that the oleanane derivatives OA and MA target the PI3K/Akt/mTOR pathway have been found in attractive breast cancer treatments.^16^ In our study the effect of OA, MA, and their combination on the expression of genes involved in the PI3K/AKT/mTOR signaling pathway in MCF7 cells was investigated. It was found that the expression of BAD, EIF2AK2, FOXO3, GSK3β, PTEN, CDKN1B, GSK3β genes after MA increased, and the AKT3, PRKCB, and TLR4 genes decreased. The expression of BAD, EIF2AK2, FOXO3, GSK3β, PTEN, CDKN1B, GSK3β genes after OA treatment increased, and AKT3, PRKCB, TLR4 genes decreased. In the combination-treated MCF7 cells, the expression of BAD, EIF2AK2, FOXO3, GSK3β, PTEN, CDKN1B, and GSK3β genes was observed to increase. AKT3, PRKCB, TLR4, IGF1 genes down-regulated.

**Figure 7:**
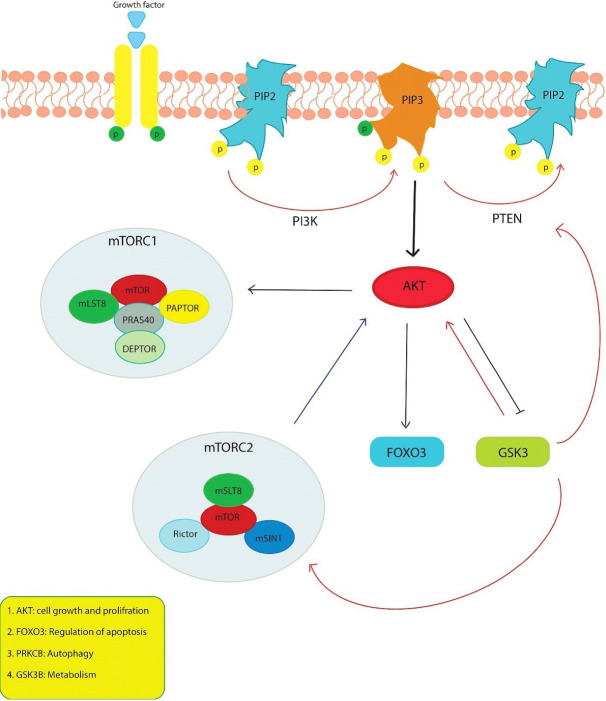
Schematic of the PI3K/AKT/mTOR pathway. Different proteins are involved in the PI3K / AKT / mTOR signal pathway so their genes can be affected by the appropriate drugs

 The PI3K/AKT/mTOR pathway is one of the effective centers for modulating proteins that regulate cell cycle and apoptosis and determine cell fate (Figure 7). Phosphorylated AKT suppresses apoptosis by playing a role in regulating downstream molecules such as BAD, caspase 9, caspase 3 and FOXO3.^22^ The expression of BAD, caspase-9 and caspase-3 at the gene and protein levels in MCF7 was examined by targeting the PI3K/AKT pathway with tanshinone, and it was found that the expression of these pro-apoptotic structures was increased.^23^ In the study conducted by Feng et al. investigating the effect of OA in lung adenocarcinomas, the natural products were found to exhibit apoptotic effect by downregulating Bcl2 and upregulating Bax and BAD.^24^ The upregulation of FOXO3, which is considered a benchmark for inhibition of the PI3K/AKT pathway and activation of AMPK, is also one of the transcription factors that regulate apoptosis by translocating from the cytoplasm to the nucleus in response to AKT.^25^ When the synthetic derivatives of OA and MA were used by Deeb et al. to target prostate cancer, FOXO3, the downstream target of AKT, mTOR and NF-KB, was inhibited.^26^ In our study, the expression of transcription factor FOXO3 increased with different treatments and the combination of OA and MA. However, some observed that their combination with each other had a synergistic effect on FOXO3 expression. A significant increase in apoptosis of MCF7 cells was observed after combination therapy with OA and MA. All this evidence suggests that the combination of the two compounds can increase the expression of FOXO3. In addition, acting on the PI3K/AKT/mTOR pathway increases apoptosis in MCF7 cells.

 AKT, one of the leading centers of the PI3K pathway, stimulates the repression of p21 and p27, which act via FOXO3 in cell cycle progression, and increases the transcription of cyclin D by inactivating GSK3 through its phosphorylation and facilitates the transition of the cell cycle from G1 to S phase.^27^ Kim et al. have claimed that breast cancer cells treated with OA arrest in the G1 phase of the cell cycle due to activation of p21 and p27 and inhibition of p-AKT and cyclin D1.^12^ In a study that investigated the effect of OA on prostate cancer in vivo and in vitro, G0/G1 arrest was due to decreased GSK3β activity mediated by AKT and decreased cyclin D expression.^13^ We found that G0/G1 arrest occurred upon treatment of natural compounds by the studies in the literature and that the combination slightly increased G0/G1 phase compared to mono-treatment. In MCF7 cells, the enhancement of G0/G1 arrest by treatment with OA, MA and their combinations could result from (i) downregulation of AKT3 gene expression, (ii) upregulation of the gene encoding GSK3β protein that blocks cyclin D coding, iii) reduction of FOXO3 expression, (iv) downregulation of the gene encoding p27.

 Besides autophagy, which is known as a cell death pathway of the II-type, it can be modulated by various signaling pathways. The main modulator for triggering autophagy is the accepted mTOR, which can be phosphorylated by the PI3K/AKT pathway and various signaling pathways.^28^ The mTOR pathway contributes to cell survival by inhibiting the formation of autophagosomes or autolysosomes in the II-type death pathway. With inhibition of mTOR, this process is reversed.^29^ LC3- II is responsible for phagophore expansion in the autophagy process, and cargo recognition by binding to the autophagosome membrane is considered a marker for autophagosome formation.^30^ Liu et al found that the expression of LC3- II was increased by modulating the mTOR and JNK signaling pathway of OA in A549 cells.^31^ Farooqi et al. used OA and MA in pancreatic cancer cells and found that OA induced protective autophagy in the cell, while MA induced autophagic cell death.^32^ The present study found that OA slightly increased autophagosomal LC3- II formation compared with the untreated group, while it increased MA. The combination contributed to II cell death compared with mono-treatment. Although the only difference between the two natural compounds in terms of chemical structure is a hydroxyl group, they can induce autophagy and apoptosis to different degrees. The coexistence of these two triterpenes and the simultaneous consumption of the same nutriment may show a strong survival inhibitory effect in the luminal A type cancer model. In agreement with the studies performed, we found that PRKCB expression decreased inversely proportional to the increasing autophagy after natural compounds and the combination treatment. PRKCB suppresses autophagy after receiving signals from IGFR1, Nox2 or ERK1/2.^33^ In our study autophagosomal LC3-II formation was induced 1.74-fold in the group treated with MA, 0.37-fold in the group treated with OA, and 3.25-fold in the combination group. There was no change in IGF1 expression in the current study when OA and MA were administered alone, but we found that IGF1 was significantly down-regulated after combination treatment. The reason for the increased autophagosome structure after two natural compounds in MCF7 cells might be the reduction of the expression of PRKCB in the downstream region of the pathway and IGF1 in the upstream region of the pathway.

## Conclusion

 The role of the PI3K/AKT/mTOR pathway is well known as a trigger for breast cancer, and it is also one of the most important pathways to establish the mechanisms of cancer resistance to treatment. Therefore, blocking various nodes in this pathway may be a suitable and valuable treatment for breast cancer. Using the combination of OA and MA decreased the resistance of cancer cells to treatment and caused cancer cells to go into apoptosis and cell cycle arrest. The combination of these two substances showed the highest synergistic effect at the lowest dose. Our study suggests that the combination of OA and MA may be a promising anti-cancer drug for treating breast cancer patients. However, more studies are needed to understand more facts. We recommend that other breast cancer cell lines be studied.

## Competing Interests

 The authors declare no conflict of interest.

## Ethical Approval

 This article does not contain any studies with human participants or animals performed by any of the authors.
